# Response to Letter to the Editor

**DOI:** 10.4274/tjo.galenos.2020.37729

**Published:** 2020-06-27

**Authors:** Banu Bozkurt, Sait Eğrilmez, Tomris Şengör, Özlem Yıldırım, Murat İrkeç

**Affiliations:** 1 Selçuk University Faculty of Medicine, Department of Ophthalmology, Konya, Turkey; 2 Ege University Faculty of Medicine, Department of Ophthalmology, İzmir, Turkey; 3 İstanbul Bilim University Faculty of Medicine, Department of Ophthalmology, İstanbul, Turkey; 4 Mersin University Faculty of Medicine, Department of Ophthalmology, Mersin, Turkey; 5 Hacettepe University Faculty of Medicine, Department of Ophthalmology, Ankara, Turkey

We thank Beuy and Wiwanitkit for their kind interest in our paper^1^ and sharing their opinions about the risk of COVID-19 infection among ophthalmologists while performing their profession. The authors stated that there are no reports in the literature about COVID-19 infection among ophthalmologists and concluded that ophthalmologists protect themselves very well or the ophthalmology services carry less risk than other medical services. However, as we mentioned in our perspective, Li Wenliang, who was the first to recognize and raise the alarm about the new disease in late December, was an ophthalmologist working in Wuhan and became infected in early January after contact with a glaucoma patient and lost his life on February 7.^2,3^ Other members of his family also suffered from COVID-19, but no other deaths were reported in the family. Recently, an article entitled “Symptomatic Covid-19 Eye Health Professionals in Wuhan Province of China” by Qiao et al.^4^ was published online on April 18, 2020. In that study, a questionnaire was sent to healthcare professionals working in ophthalmology departments in hospitals in the Wuhan province to understand the incidence of symptomatic COVID-19 among eye professionals. The survey was sent to 28 eye professionals with symptomatic COVID-19 diagnosed through February 29, 2020 and 90 control participants randomly selected within each ophthalmology department where case(s) were identified. Among 28 eye professionals from 10 hospitals who contracted COVID-19 with pulmonary symptoms, there were 14 ophthalmologists, 12 ophthalmic nurses, and 2 ophthalmic technicians. Two participants could not answer the questionnaire; one died and another remained intubated through data collection. All 3 deaths were ophthalmologists who had worked in the same hospital. Only 5 eye health professionals with confirmed COVID-19 (17.9%) had family members with symptomatic COVID-19, which means the virus was not transmitted from family members in most of the patients. The overall incidence of symptomatic COVID-19 among eye professionals across 10 hospitals was found to be 2.52%. Extrapolated from data available from the Chinese Red Cross Foundation and Wuhan Health Commission,^5,6^ the estimated COVID-19 incidence among all healthcare workers in the 10 hospitals was 2.27% (713 of 31367), which means the risk of symptomatic COVID-19 is similar among ophthalmologists and other health professionals. Based on these findings, we cannot say that the disease is uncommon among ophthalmologists or that ophthalmology practice carries less risk than other medical practices.

Qiao et al.^4^ also reported that after implementing restrictions such as eye examinations and surgeries conducted only in emergency circumstances, city lockdown, use of appropriate personal protective equipment during clinical practice, and careful hand hygiene, the number of professionals infected by SARS-CoV2 decreased remarkably, similar to our recommendations for ophthalmologists.^1^
